# Developments in Transduction, Connectivity and AI/Machine Learning for Point-of-Care Testing

**DOI:** 10.3390/s19081917

**Published:** 2019-04-23

**Authors:** Shane O’Sullivan, Zulfiqur Ali, Xiaoyi Jiang, Reza Abdolvand, M Selim Ünlü, Hugo Plácido da Silva, Justin T. Baca, Brian Kim, Simon Scott, Mohammed Imran Sajid, Sina Moradian, Hakhamanesh Mansoorzare, Andreas Holzinger

**Affiliations:** 1 Department of Pathology, Faculdade de Medicina, Universidade de São Paulo, São Paulo 05508-060, Brazil; 2 Healthcare Innovation Centre, Teesside University, Middlesbrough TS1 3BX, UK; z.ali@tees.ac.uk (Z.A.); S.M.Scott@tees.ac.uk (S.S.); 3 Faculty of Mathematics and Computer Science, University Münster, 48149 Münster, Germany; xjiang@uni-muenster.de; 4 Department of Electrical and Computer Engineering, University of Central Florida, Orlando, FL 32816, USA; reza@eecs.ucf.edu (R.A.); Brian.Kim@ucf.edu (B.K.); moradian@knights.ucf.edu (S.M.); Hakhamanesh.Mansoorzare@UCF.EDU (H.M.); 5 Department of Electrical and Computer Engineering and Biomedical Engineering, Boston University, Boston, MA 02215, USA; selim@bu.edu; 6 IT—Instituto de Telecomunicações, 1049-001 Lisbon, Portugal; hugoslv@gmail.com; 7 Department of Emergency Medicine, University of New Mexico School of Medicine, Albuquerque, NM 87131, USA; JTBaca@salud.unm.edu; 8 Department of Upper GI Surgery, Wirral University Teaching Hospital, Wirral CH49 5PE, UK; misajid14@gmail.com; 9 Institute for interactive Systems and Data Science, Graz University of Technology, 8074 Graz, Austria; andreas.holzinger@medunigraz.at; 10 Institute for Medical Informatics, Statistics and Documentation, Medical University of Graz, 8036 Graz, Austria

**Keywords:** POCT, deep learning, artificial intelligence, photonics, mobile phone, microfluidics

## Abstract

We review some emerging trends in transduction, connectivity and data analytics for Point-of-Care Testing (POCT) of infectious and non-communicable diseases. The patient need for POCT is described along with developments in portable diagnostics, specifically in respect of Lab-on-chip and microfluidic systems. We describe some novel electrochemical and photonic systems and the use of mobile phones in terms of hardware components and device connectivity for POCT. Developments in data analytics that are applicable for POCT are described with an overview of data structures and recent AI/Machine learning trends. The most important methodologies of machine learning, including deep learning methods, are summarised. The potential value of trends within POCT systems for clinical diagnostics within Lower Middle Income Countries (LMICs) and the Least Developed Countries (LDCs) are highlighted.

## 1. Introduction

Poor health conditions shorten peoples’ lives and undermine their quality of life. These conditions also limit economic and social development by reducing ‘human capital’ and generating health costs. More broadly, long and healthy lives are important indicators for societal well-being. Within industrialized nations there is a challenge of increasing demand, typically arising from an aging population, rising costs and decreasing resources. Within Lower Middle Income Countries (LMICs) and the Least Developed Countries (LDCs) there is an enormous lack of resources. Here disruptive innovations may have the potential to improve health outcomes, lower costs and improve access. Particularly, there is a need to move health care away from symptomatic treatment of diseases by blockbuster drugs and towards a more predictive, preventive and personalized medicine.

For most chronic disorders—such as cancer, metabolic disease, cardiovascular disease, diabetes and dementia the disease process starts decades earlier before it appears symptomatically. Identifying individuals at the earliest stage of their disease will completely change the therapeutic paradigm and transform the way that health care is delivered so that it is more focused on sustaining health rather than treating late stage-patients with symptomatic disease. There is also an opportunity of disruptive innovation through development of new models of person-centered community-based health delivery which allow decentralization from traditional secondary care providers, such as hospitals, to care delivered within primary and tertiary care settings (e.g., care homes).

Point-of-Care Testing (POCT) that is easy to use and interfaces with medical records or a broader health surveillance system, has the potential to dramatically alter disease burdens through home-testing or self-testing. Patients who may avoid seeking medical care for a stigmatized condition could perform testing without having to report to a public health center or physician’s office [1]. Simple, standard interfaces for POCT results, could overcome some of the traditional barriers to widespread adoption by allowing for testing in the most convenient and medically appropriate location, at the point of need.

## 2. Patient Need for POCT

### 2.1. Infectious Diseases

POCT can play an important role in both the diagnosis and management of specific diseases. POCT for the rapid identification of specific pathogens [2] is ongoing. The need for POCT for the early identification and management of sepsis is particularly important since this is difficult to diagnose and the mortality increases by an average of 8% for every undiagnosed hour [3]. In the hospital setting, various tests and investigations are undertaken when suspecting sepsis in both adults and children: these include vital signs checks (observations of temperature, blood pressure, respiratory rate, pulse oximetry oxygen saturation, heart rate and responsiveness). A full set of routine blood tests can include blood count, a chemistry profile including urea and electrolytes, C-Reactive Protein (CRP), and glucose, and a coagulation screen. More than 170 biomarkers have been reported in the literature for the diagnosis of sepsis. However, only the most common biomarkers, such as CRP or procalcitonine, can typically be analysed within centralised hospital laboratories. The testing of the other biomarkers typically requires more specialised laboratories. Other tests can include arterial or venous blood gas samples, urine output recorded and urine sample dipstick test and microbiological culture, aerobic and anaerobic microbiological blood cultures, swab of suspected wound or respiratory tract/gynaecological swabs, chest radiograph.

Some of these can be tested immediately to provide results (at the patient’s bedside), others require sampling of bodily substances/fluids which can either be taken to a local analyser or sent to laboratories (these may be onsite or require further delivery to more specialist laboratories elsewhere offsite). Results may be published to patient’s electronic records as soon as possible. Some of the requested tests may be marked urgent or routine, as necessary. Bedside glucose tests, vital signs temperature probe, pulse oximetry or electronic blood pressure cuffs will often provide immediate results. Arterial or venous blood gas samples can be difficult to acquire if there is difficulty with gaining vessel access (sometimes alternative sampling techniques including femoral stab, or ultrasound-guided sampling is necessary). The local availability of analysers can, however, lead to results within minutes, including true oxygen and carbon dioxide saturations, pH, lactate and Electrolyte levels. Similarly urine samples may be tested with a dipstick for common abnormalities (protein, leukocytes, blood, glucose, nitrates), or urine test for pregnancy, prior to being sent for full investigation in the laboratory. Blood or swab cultures taken will require 5–10 days incubation; growth of any organisms and their sensitivities/resistance to various antibiotics will guide management and assist in identifying appropriate treatment(s). Currently, certain patients found to be positive with or exposed to certain microorganisms (’superbugs’) may need more treatments by nursing staff to improve sanitation or nursing in isolation; examples include patients positive for Methicillin-resistant Staphylococcus aureus (MRSA), Vancomycin-resistant Enterococci (VRE), and Carbapenemase-producing Enterobacteriaceae (CPE), among others.

Sexually transmitted infections (STIs) are among the most common acute conditions worldwide. In 2012, there were 357.4 million new global cases of four common curable STIs: chlamydia (130.9 million cases); gonorrhoea (78.3 million cases); syphilis (5.6 million cases) and trichomoniasis (142.6 million cases) [4]. Although STIs are not normally fatal they do represent a significant burden of diseases and they can lead to complications such as pelvic inflammatory disease, ectopic pregnancy and infertility. STIs can increase infectiousness of a susceptibility to HIV and in pregnancy they can cause fetal or neonatal death. Chlamydia is globally the most common bacterial STI and causes reproductive complications in women. The LMICs and LDCs have the majority of global incidents of STIs, but the health systems are less well resourced to manage these. POCT could play an important role in supporting the management of conditions for individual patients and also provide wider control of STIs within developed countries, LMICs and LDCs.

According to the Centers for Disease Control and Prevention (CDC), in the United States alone drug-resistant bacteria cause at least 23.000 deaths and 2 million infections every year. This is therefore a public health problem, requiring innovative POCT approaches that can contribute to helping characterise the development and spread of resistant bacteria [5,6]. This can have a transformative role in the treatments administered, constituting one step further in the path of personalised medicine. By allowing the rapid detection of infectious pathogens and resistance factors, practitioners can for example reduce unnecessary administration of antibiotics, contributing to alleviate the problem of antibiotic resistance.

Respiratory tract infections (RTIs) are one of the problems that can be caused by a variety of bacterial and viral pathogens. Worldwide, they are the second greatest cause of morbidity and mortality [7]. RTIs are the most common infections for those that are immuno-compromised. There is now also concerns about the number of infections caused by antimicrobial resistant (AMR) bacteria as well as community and hospital acquired infections, for example pneumonia. New lethal viruses and bacteria causing RTIs with epidemic potential have emerged over the last decade. These include severe acute respiratory syndrome coronavirus (SARS-CoV), swine-origin influenza A, multi-drug resistance tuberculosis and multi-drug resistance gram negative bacteria, for which there are very few effective therapy options.

The key challenge for effective treatment of RTIs in a variety of different healthcare settings is for fast, sensitive and specific identification of pathogens as well as antibiotic resistance profiles. Also, it would be beneficial to determine whether a pneumonia was Community-acquired or Hospital-acquired (there are cases where this is still unclear): this can have a large impact on treatment regimen. If Hospital-acquired, then treatment with intravenous antibiotics initially is more beneficial. RTIs are the most common infections encountered within primary care and there is evidence that people presenting with acute uncomplicated RTIs will commonly receive antibiotics despite most RTIs being viral [8]. There is therefore a clear need for POCT that can differentiate RTIs within primary care and reduce unnecessary antibiotic prescribing as part of AMR stewardship.

### 2.2. Non-Communicable Diseases

Non-communicable diseases with the highest health burdens include cardiovascular diseases, cancer and diabetes. The number of POCT that are commercially available for non-communicable diseases is limited and reflects the challenge of measuring low concentrations of protein biomarkers in a variety of different biological samples, including blood, serum, urine and saliva. The key cancer and cardiac biomarkers, as well as their normal values in blood, are given in Table 1. PSA is the most common tumour marker for prostate cancer. IL-6, an interleukin, is overexpressed in several different cancers including prostate cancer, as well as head and neck squamous cell carcinoma. The interleukins are part of the cytokine family and more generally play an important role in the inflammatory response of diseases such as rheumatoid arthritis, cardiovascular diseases (CVD), diabetes and Alzheimer’s disease. The MMP family are part of the zinc-dependent endopeptidases, where MMP-2 is key in tumour growth, invasion and metastasis and MMP-3 is used to diagnose and monitor diseases such as head and neck squamous cell carcinoma and adrenal tumours. The alpha-fetoprotein, an oncofetal glycoprotein, is the most important for liver cancer tumour marker. CEA, a glycoprotein, is raised with inflammation or tumours in any endodermal tissue, including the gastrointestinal tract, respiratory tract, pancreas and breast, and can be used for diagnosis of lung cancer, ovarian carcinoma and breast cancer. The cancer antigen 125 (CA-125) is used for monitoring ovarian, breast and uterine cancer.

CVDs and coronary heart disease are globally among the leading causes of ill health, invalidity and mortality. Troponin-I is most sensitive for myocardial tissue damage [9]. There are several cardiac biomarkers, these can sometimes require 3-hourly or 6-hourly repeat blood tests, checking for dynamic rise and can be used to rule-out or confirm Acute Coronary Syndrome (ACS). D-dimer blood tests can be used to help rule out suspected Deep Vein Thrombosis (DVT) or Pulmonary Embolism (PE), but it suffers from a high false positive rate that may lead to unnecessary or costly advanced imaging.

Similarly, there are certain Human Leukocyte Antigens (HLA) that are associated with specific autoimmune and immune-mediated diseases, such as reactive arthritis, skin lesions, systemic lupus erythematosus (HLA-DR2), rheumatoid arthritis, diabetes mellitus type 1 (HLA-DR3) and ankylosing spondylitis (HLA-B27). HLAs are also very important in organ transplant rejections, where there may have been inadequate matching. We might include tests for Erythrocyte Sedimentation Rate (ESR), Rheumatoid Factor (RF), Antinuclear Antibody test (ANA) and, then potentially more specific autoantibody tests when investigating chronic or autoimmune diseases. There are also many chronic or autoimmune diseases linked to genetic mutatations, or even rare conditions such as Gorlin Syndrome [10].

POCT with a higher level of connectivity and data analytics could dramatically change the diagnosis and management of both communicable and non-communicable diseases. Moreover, incorporation of findings from the human genome project with decentralized POCT could dramatically change our understanding of disease epidemiology and population health.

## 3. Developments Towards Portable Diagnostics

Diagnosis of disease is commonly carried out by the quantification of DNA, RNA and proteins or other biomarkers using a variety of biochemical assays in a centralised laboratory setting and this remains powerful in accurately detecting diseases. The drawback of using large highly automated floor standing analysers in a centralized laboratory setting is that the instrumentation used is expensive and it requires operation by staff with specialized technical skills. Moreover, it can take a considerable amount of time for the assay to be performed, particularly if there is a requirement for batches of samples to be gathered before measurements can be made. More recent developments are focused toward automating and miniaturizing the traditional biochemical assays, such as enzyme linked immuno-sorbent assay (ELISA) and polymerase chain reaction (PCR) to increase their applicability and accessibility [11].

Microtiter plate assays are the gold standard for immuno-assays and are performed in an array of wells of varying density and volume as part of a microplate. The 96-well plate (well volume 530 μL) is fairly ubiquitous and the 384-well plate (well volume 149 μL) is common. There is a trend towards higher-density, lower-volume well plates (with the 1536 well plates having a volume of 10 μL). The smaller volumes are crucial because they allow a lower volume of sample, solvents and reagents which greatly reduces the cost of the assay. The most commonly used immuno-assay format is the ELISA, which typically captures an analyte (antigen) between two antibodies and upon which, one antibody is labelled with an enzyme. The enzyme enables a signal enhancement by a factor of 100,000 by converting a substrate into a detectable signal. For signal generation, the substrate can be converted either into an absorbing dye (detected by absorption of transmitted light), into a fluorescent dye (detected via a fluorescence reader) or into a light emitting reaction (detected as luminescence). Fluorescence and luminescence are more sensitive as compared with absorbance but are also more expensive and can involve more complex protocols. The choice of approach for signal generation and detection is therefore generally dependent on the sensitivity and cost requirements.

Microtitre plate assays and ELISA are widely used for clinical analysis but they can typically take several hours and the complex assay protocol involves skilled personnel, as well as generally expensive automated analysers. The demand for portable diagnostics is, however, that they should be fast, reproducible and are able to be operated by untrained users. Lateral flow assays are widely used as a robust, simple and low cost analytical assay. They are most commonly used for simple Yes/No answers such as testing pregnancy or drug abuse in a qualitative or semi-quantitative manner. There is now greater effort in the development of quantitative lateral flow assays. Lateral flow assays typically use antibody coated microparticles, which bind the analyte directly from the sample and are then enriched via binding against a second antibody in a target zone. This obviates the need for complex and costly enzymatic signal enhancement used within microplate ELISA.

After the lateral-flow assays, simple electrochemical enzymatic assays are the most commonly used for quantification of small molecules, which can be detected quantitatively in redox-reactions. The most widely used application for these has been blood glucose monitoring for diabetes with a glucose oxidase enzyme acting as a catalyst for the conversion of a glucose substrate to a gluconolactone product and with amperometric detection [12,13]. More recently, there has been increasing interest in the use of impedimetric sensing as a label-free approach for monitoring ligand receptor binding [14,15]. Use of a label on a biomolecule has the drawback of altering the binding properties of the biomolecule. A label can allow higher sensitivity and or selectivity but this is typically at the expense of additional cost, time and sample handling. Impedimetric sensing is intermediate between equilibrium and dynamic electrochemical methods. In an equilibrium method such as potentiometry, there is no current flow and the cell potential is related to the concentration of the analyte. In a dynamic method such as voltammetry, the potential is controlled and the current is measured as a function of the potential. In impedimetric analysis, a small ac potential is applied, superimposed on a preset or offset voltage and, the time dependent current is measured which allows calculation of the impedance. In dynamic methods, a current is allowed to flow and so there is a change in the sample constituents, which is not the case for equilibrium measurements. In impedimetric measurements a small current is allowed to flow, but this is then quickly reversed so that there are no significant changes in the sample. Impedance sensing is typically carried out using interdigitated electrodes where the electrode width and separation determines the penetration depth of the electric field. When a ligand binds to a receptor, the change in the electric properties leads to a change in the impedance which provides a direct electrical signal for the binding event. A key advantage is that the electrode configuration can be designed such that the electrodes scan just above the electrode surface to the depth of the bound ligand-receptor, which would then minimise any interferences from the bulk of the solution [16]. Impedimetric sensing is also attractive since micro and nanofabrication techniques would allow development of the miniaturised transducers in a convenient manner. The potential of fabricating an impedimetric array in a polymer microfluidic cartridge has been demonstrated as a potential aid for diagnosis of Deep Vein Thrombosis/Pulmonary Embolism (DVT/PE) and towards low cost point-of-care diagnostics [17] An impedimetric array within a microfluidic cartridge could allow multiple measurements of a single biomarker or alternatively measurements of multiple biomarkers within the same microfluidic cartridge.

For detection of pregnancy or drug-abuse, a simple Yes/No answer using “visual” readout is sufficient, but the majority of clinical assays require quantification with defined handling for the applied volumes, incubation time and analysis of the generated signal. In recent years, there has been great interest in the use of “Lab-on-a-chip” or “microfluidic” systems which can allow fine control of fluidic operations with control elements, different detection elements and integrated bioreagents for internal standards and for assay. These have been developed for a variety of applications including diagnostics, bioprocessing [18,19] and cytotoxicity testing as part of drug discovery [20]. The Lab-on-a-chip term is typically used when the device is performing a specific application whereas microfluidics is a more general term which applies for miniaturised fluidic volumes either as passive—within a container—or active so that the fluidics might be driven. Microfluidic devices can potentially fill the technological gap between simple “lateral flow” POC tests and sophisticated laboratory-based analysers. Specifically, microfluidics offer advantages of controlled microenvironment with automated and highly reproducible operations, reduced energy consumption as well as reduced amount of reagents and shorter assay times [21]. Microfluidics offer the potential for the development of devices as simple “sample in—answer out” with a minimum of handling steps, ease of use and a high level of robustness and reliability. There are considerable challenges in the widespread use and practical utility of microfluidic devices including: a reduction in the chip costs, which remain high; reducing the cost and size of optical readout systems; ease of fluidic control; and on-chip storage of reagents.

New progress in portable diagnostics is focused on lowering the cost, simplifying the instrumentation and, thus increasing the accessibility of diagnostic tools to the wider population. 3D printing technology has been used to significantly lower the cost and time of developing novel diagnostics devices compared with conventional manufacturing approaches [22,23,24,25,26,27]. 3D printing technology has been used for incorporation of integrated valves [25,26,27] as well as for point-of-care colorimetric analysis [22].

## 4. Novel Photonic Systems

### 4.1. Cavity Enhanced Absorption Spectrometry (CEAS)

CEAS has been shown to have higher sensitivity than conventional single pass absorption detection. In CEAS, high sensitivity is achieved through increasing the pathlength by locating the sample between two highly reflective dielectric mirrors. The light reflects between the two mirrors to form an optical cavity which magnifies the optical absorption effect. A very small amount of light exits from the mirror and this is measured to give the CEAS signal [28,29]. The CEAS technique has the potential to approach the sensitivity of fluorescence based detection but with the advantages of lower reader cost, due to fewer optical elements—and being label-free. Overall, CEAS offers the potential for higher sensitivity; wider applicability; less complex assay protocols with the associated lower assay costs; and potentially no interference of the native ligand receptor binding arising from a label. Early studies using a low cost visible light-emitting diode (LED) as a light source and a 2-mm cuvette using a range of samples at visible wavelengths showed a 50-fold improvement in sensitivity compared to conventional single pass uv-visible spectroscopy [30]. Subsequent work demonstrated that CEAS could be used as a detector for High Performance Liquid Chromatography (HPLC) measurements. This involved placing a HPLC flow cell (70 microlitre) into an optical cavity. A 50–70-fold improved sensitivity was demonstrated over a conventional HPLC instrument [31]. A 39-fold increase in sensitivity has been demonstrated for a 96 well microtiter plate commercially available osteocalcin ELISA and a 115-fold increase in sensitivity for a commonly used ELISA colorimetric reaction of streptavidin-horseradish peroxidase (STREP-HRP) with Tetramethylbenzidine (TMB) [32]. CEAS has also been demonstrated for stopped flow kinetics [33].

### 4.2. Plasmonics

**Figure 1:**
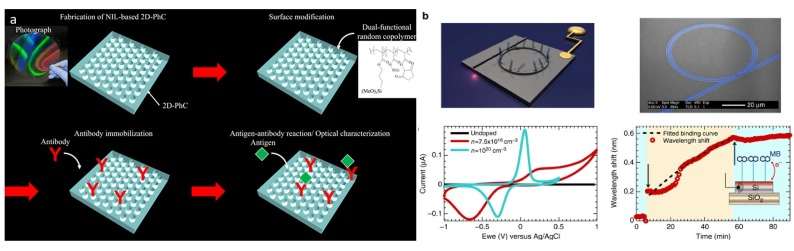
Recently developed sensors for molecular diagnostics. (**a**) Photonic crystal structure based on nanoimprint lithography for immunoassay. (**b**) An electrophotonic sensor that combines electrochemical and photonic characterization. Reprint from [38,39].

Recent sensor developments using immuno-assays continue to yield higher sensitivity [34,35,36]. Unlike conventional ELISA, plasmonic ELISA uses the aggregation of gold nanoparticles as the reporter when the biomarker is present that exhibits shifted extinction characteristics, thus a change in observable colors [35]. Similarly, a method has been reported to use the reduction of silver ions onto gold nanoparticles to amplify the signal originating from immunosandwich for the simultaneous and rapid diagnosis of HIV and syphilis [34]. This technique was able to measure very low concentrations, as low as 1 Ag/mL. Photonic crystal gratings are proposed as a new sensor for biosensing applications [37]. The resonance characteristic of photonic crystal gratings is sensitive to small changes in dielectric permittivity caused by biomarker bindings (Figure 1). Combining the photonic crystal grating with an immunoassay, C-reactive protein, which is produced in the presence of inflammation, can be detected at a detection limit of 12.24 pg/mL [38]. Multiplexed sensors can diagnose more than one infectious disease by detecting various biomarkers. An electrophotonic sensor was able to profile biomarkers by combining electrochemical and photonic characterization [39]. Using the selective chemical functionalization, the electrophotonic sensor array can identify different binding events. A barcoded paper-based analytical device was used to show a multiplexed detection of eight antigen types using lateral flow [40].

### 4.3. Digital Microarrays—Interferometric Reflectance Imaging Sensor (IRIS)

Disease diagnostics have been evolving through the synergistic collaboration of medicine with engineering and science. With the advent of the measurement/sensing technologies that provided the ability of detecting trace substances in bodily fluids such as blood, urine, and cerebrospinal fluid in vitro Diagnostics (IVD) have become a cornerstone of clinical practice. Solid phase immuno-assays such as ELISA [41] have long been established and are used extensively for diagnosis. The vast majority of sensing technologies used for molecular diagnostics are ensemble measurements, in other words they are analog. These immuno-assays have sensitivities in the picomolar range [42]. However, the serum concentrations of most protein biomarkers in the early stages of infection [43] are in the femtomolar range. To achieve the desired 3-order-of-magnitude improvement, a new class of biological sensing technologies have emerged relying on single molecule counting or digital detection, an approach that provides resolution and sensitivity not obtainable with ensemble measurements. Digital detection is a disruptive technology that can provide the necessary improvement in sensitivity. Furthermore, it is much easier to measure the presence or absence of signal than to detect the absolute amount of signal as it has long been recognized in communications and data recording. The advent of digital audio recording simplified the requirements of readout and allowed for reproduction of highest quality music without the expensive Hi-Fi equipment. Similarly, digital detection may lead to the most advanced disease diagnostic tools to become available at a low cost and at the point-of-need.

Most of the digital detection techniques are based on particle confinement in an isolated microenvironment, allowing for the necessary signal-to-noise ratio [44]. Among these sample compartmentalization techniques, the most notable one is digital PCR [45]. Solid phase and microarray-based techniques have the promise of low-cost and point-of-need operation. DNA and protein microarrays are high-throughput and allow the simultaneous quantification of tens of thousands of different biomolecules. Their sensitivity, however, is often insufficient as a reliable diagnostic tool. This practical limit is not imposed by the microarray format itself, but rather the sensitivity of conventional methods involving fluorophores as labels and fluorescence readers. Recent advances with Interferometric Reflectance Imaging Sensors (IRIS) [46] show their ability to detect single nanoscale particles by enhancing the elastic optical scattering signatures in a wide-field optical imaging format [47]. In single-particle modality of IRIS (SP-IRIS), the interference of light reflected from the sensor surface is modified by the presence of particles producing a distinct signal that reveals the size of the particle. Identification of virus particles in complex samples in multiplexed format have been demonstrated [48]. Size discrimination of the imaged nanoparticles (virions) allows differentiation between modified viruses with different genome lengths and, facilitates a reduction in the counting of non-specifically bound particles. This technique has been adapted to a closed-system to allow real-time particle detection in complex mixtures [49]. For this application to detect viral hemorrhagic fever viruses, biosafety was achieved by having a sealable sample addition port. The platform demonstrated capability of identifying virus binding in a 20-min experiment. Sensitivity for this rapid test was comparable to laboratory-based assays such as ELISA and plaque assay. These results demonstrate a digital detection platform that has promise for rapid, multiplexed, sample-to-answer diagnostic technology.

**Figure 2:**
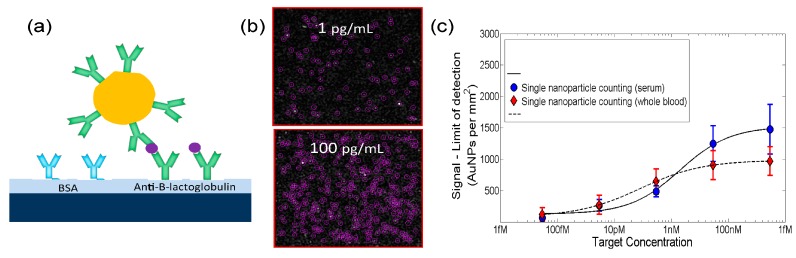
(**a**) Conceptual representation of SP-IRIS single molecule counting assay for protein biomarker detection; (**b**) Representative images at 1 pg/mL and 100 pg/mL target concentration; (**c**) Dilution curve for β-lactoglobulin in unprocessed serum and whole blood. Adapted with permission from Ref. [51]. Copyright (2013) American Chemical Society.

While SP-IRIS can detect individual biological nanoparticles such as viruses and exosomes [50] without labels, it utilizes nanoparticles as labels for “digital” detection of individual protein and nucleic acid molecules, as illustrated in Figure 2 [51]. SP-IRIS can detect Au nanoparticles as small as 20 nm, which is only about twice the hydrodynamic diameter of an antibody, allowing this information to serve as an identifier of the biomolecule attached to the nanoparticle. Using this approach, the detection of protein biomarker, β-lactoglobulin, has been demonstrated in unprocessed serum and human whole blood with detection limits of respectively 60 aM and 500 aM. Direct detection of protein biomarkers with attomolar sensitivity without the need for sample preparation can allow performing diagnostic tests at the point of care. Quantitation of allergen-specific IgE from unprocessed finger-prick volume of human blood has also been shown [51]. More recently, development of a digital microarray has been reported using functionalized gold nanorods as single-molecule labels and an interferometric scanner, which can rapidly enumerate individual nanorods by imaging them with a 10× objective lens [52]. By combining single-nanoparticle enumeration and ensemble measurements of spots when the particles are very dense, this system achieves a dynamic range of about 6 order-of-magnitude directly from a single scan. As a proof-of-concept a digital protein microarray assay has been demonstrated for detection of hepatitis B virus surface antigen in buffer with a limit of detection of 3.2 pg/mL. More broadly, the technique’s simplicity and high-throughput nature make digital microarrays a flexible platform technology with a wide-range of potential applications in biomedical research and clinical diagnostics [52].

**Figure 3:**
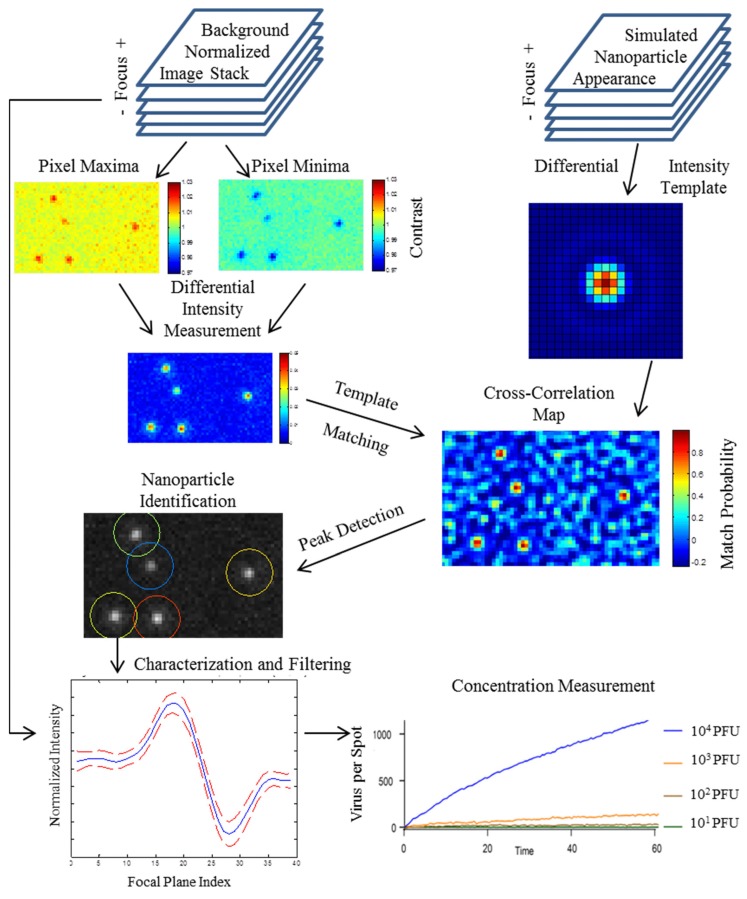
Block diagram of algorithm for nanoparticle detection and counting using z-stacks of incrementally defocused images. Adapted with permission from Ref. [53].

Based on the increased fidelity of read-out, digital detectors offer the potential to fill the diagnostic gap between ultrasensitive molecular amplification tests and qualitative POC tests, by promising both direct and sensitive measurement of health biomarkers. Despite the potential for very high sensitivity demonstrated by SP-IRIS in a simple and inexpensive microarray format, the robustness of the image acquisition and particle-counting software has been a major challenge. Key challenges in translating SP-IRIS (from a laboratory instrument requiring manual operation by a skilled operator to an automated tool for diagnostic applications) includes stringent calibration and characterization requirements. As a first step, it is crucial to develop a robust image acquisition and analysis technique. It has been discovered that the change in intensity varies for the signature of a nanoparticle over the focal plane, or differential intensity is considerably more predictable than its specific appearance in any single image. Thus, the nanoparticle response generated by the calculation of differential intensity collapses into a consistent profile, enabling the use of simple template matching methods. As illustrated in Figure 3, an algorithm has been developed for the robust measurement of the concentration of surface-bound nanoparticle populations, regardless of heterogeneity in size and axial offset [53].

The nanoparticle defocus images result in a particle-specific response in interference-enhanced wide-field nanoparticle imaging as in SP-IRIS. Thus, this particle specific signature provides unique opportunities for nanoparticle classification (for example, size, type and shape) upon detection. A model-based supervised learning algorithm has been combined with a wide-field common-path interferometric microscopy method to achieve accurate nanoparticle classification [54]. The classification schemes have been verified experimentally by blindly detecting gold and polystyrene nanospheres, and then classifying them in terms of type and size. It is clear that supervised machine learning techniques will be indispensable tools that will enable robust and objective analysis for POCT digital sensors that are based on detection of biological nanoparticles or nanoparticle labels attached to individual protein and DNA molecules.

## 5. Mobile Phone Reader and Device Connectivity for POCT

### 5.1. Components of Mobile Phones Used in POCT

Rapid technological improvement in mobile phone connectivity and functionality as well as their increased global market penetration has opened new avenues in biomedical research, education and applications. These improvements have been accompanied by an explosion in new sensing modalities that are enabled by batch-fabrication of complex transducers on a single chip [55,56,57,58]. The growth of such complementary technologies has resulted in new and exciting mobile phone-based point-of-care sensors. Mobile phone functions that are key to the development of point-of-care systems include: sensors (camera, microphone, etc.), communication (Bluetooth, Wi-Fi, cellular, etc.), sophisticated multi-touch user interface, enhanced battery, huge data storage and processing capacities. We describe here the mobile phone functions as they relate to point-of-care systems and review recent developments in detection of infectious diseases with a focus on point-of-care systems developed for in-field use.

Mobile phones house an array of sensors ranging from cameras operating in visible and infrared spectrum to microphones and proximity sensors. Each sensor is a valuable source for collecting information that can be tapped into for point-of-care applications. Practically all newer generations of mobile phones contain built-in high resolution cameras that are capable of detecting amplitude (brightness) and wavelength (shade of color) of incident light with high accuracy. They operate by projecting incident light using a lens on a rectangular array of micron scale photo-sensors that detect light in the visible spectrum. Following data processing and enhancement, information from a single or a group of adjacent photo-sensors is stored as a single pixel in the final image.

In Liao et al. (2016) [59], researchers developed a molecular diagnostic point-of-care system that processed a sample using the mobile phone’s flashlight to excite fluorescence dye and consequently the camera was utilized to measure average fluorescence reaction light intensity in real time. The system used a water triggered exothermal reaction to regulate temperature and took advantage of a custom-made microfluidic chip for amplification. This system was reportedly capable of consistently detecting as few as 100 copies of herpes simplex virus type 2 per sample. Infrared cameras operate using the same mechanism as visible light cameras, with the key difference being that the photo-sensors are made sensitive to infrared light (longer wavelength). Infrared cameras detect temperature profile of objects and, due to the higher penetration depth of infrared light compared to visible light, they have the capability of visualizing subsurface features. Infected wounds exhibit a characteristic temperature profile even in cases where the infection exists in the underlying tissue of a closed wound. Researchers have developed a non-invasive point-of-care system that analyzes thermal images produced by an auxiliary infrared camera of a mobile phone and successfully diagnosed infected closed wounds [60].

Presently, microphones used in mobile phones are almost exclusively silicon micro electro mechanical system (MEMS). In MEMS microphones, a suspended micro-structure deflects as a function of the amplitude and frequency (pitch) of incoming acoustic pressure waves. This deflection is then converted to an electric signal using either capacitive or piezoelectric transduction. The airflow into and out of the respiratory system generates acoustic waves that can be detected by mobile phone microphones making the respiratory track a test subject for this class of point-of-care systems. An example of such a system correlates the spectrum of respiration sound to flow rate of air, which can then be used for diagnosis of respiratory conditions, including asthma, chronic obstructive pulmonary disease (COPD), and cystic fibrosis [61]. Quality of measurement is heavily reliant on signal processing techniques to reduce the effects of environmental noise and dependence on microphone distance. Goel et al. (2016) [62] improved the accuracy of air flow rate measurement using a whistle that changes in pitch as a function of flow rate in combination with a mobile phone microphone. Improved mobile phone communication technology and infrastructure has made it possible for fast, reliable, secure, accessible data transfer between mobile phone-based point-of-care systems and the network. At its most basic form, communication between mobile phone-based point-of-care systems and the network, enables transmission of the findings. However, given the reality of current 4G and Long-Term Evolution (LTE) technology and next generation 5G connectivity, mobile phone-based point-of-care systems are no longer limited to the in-built mobile phone data processing and storage capabilities. They have near real-time access to the significantly larger computational and data storage capacity of remote servers. In addition to the above functionalities, mobile phones offer researchers a sophisticated and accessible hardware platform for software implementation, user interaction and an energy source for low power point-of-care systems.

**Figure 4:**
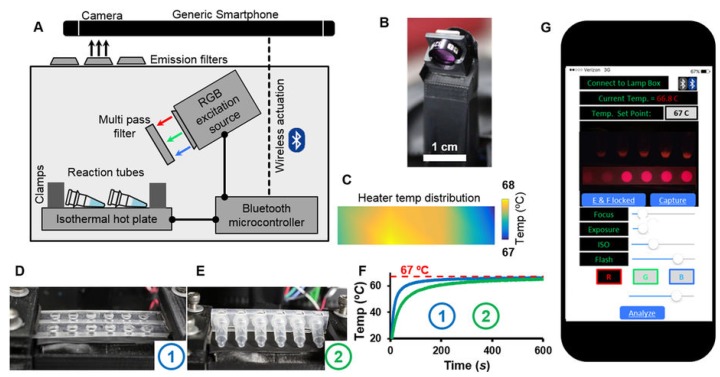
Zika virus sensor. (**A**) Test tubes placed on Isothermal hot plate are imaged using the smartphone camera with the LED acting as a light source. (**B**) Smartphone application interface. (**C**) Measured heat map of surface temperature indicating uniform heating with less than 1 ${}^{{}^{\circ}}$C temperature variation. (**D**) Off the shelf PCR polypropylene tubes. (**E**) Custom made laser-cut reaction wells. (**F**) Measurements show improved thermal management with custom reaction wells. (**G**) Smartphone wirelessly controls the heating lamp and excitation source and is also responsible for capturing and analyzing illuminated reagents. Reprint adapted with permission from [63].

In Priye et al. (2017) [63] a mobile Zika virus detection unit was developed for in-field use (Figure 4). Utilizing fluorescence signals, Zika viruses were detected from crude human sample matrices including blood, saliva, and urine. Samples collected in plastic tubes were placed on an isothermal heater and excited from LED sources. The use of crude samples, with minimal required preparation, drastically improves the capability of rapid outbreak response teams. In addition to Zika virus, the sensor is capable of rapid detection of dengue and chikungunya viruses. This system is relatively inexpensive ($100) and can detect Zika, dengue, and chikungunya viruses within 30 min.

With the aim of reducing the timescale and complexity required for the detection of the Zika virus in mind, Chan et al. (2016) [64] created a portable low-cost mobile phone camera-based sensor, with the assistance of a commercial 3D printer as a converted automated robotic arm. The sensor operates similarly to the gold standard for Zika virus detection and utilizes reverse transcription recombinase polymerase amplification (RT-RPA)/reverse transcription polymerase chain reaction (RT-PCR). Once amplification is completed, blue LEDs excite the samples and the resulting fluorescent emission signals are measured using a mobile phone camera. This rapid response sensor was found to be highly selective for Zika virus as the RT-RPA and RT-PCR assays do not cross-react with dengue and chikungunya viral RNA. The measurement is reported to take approximately 30 min and to be capable of simultaneously testing 12 urine samples.

Improved mobility of sensors can greatly improve the impact of any outbreak response. By using a consumer grade drone to deliver the sensor unit, Priye et al. (2016) [65] utilized a charging unit (used for mobile phones) to power the sensor. Moreover, the drone’s rotor powers a centrifuge that is used for preparing the samples. This system uses a mobile phone camera with an image analysis app for time resolved fluorescent detection and quantification. The sensor cost was reported to be $50. Interestingly, it was reported that tests can be performed mid-flight, thus further improving the response time.

User convenience is an important advantage of mobile phone-based biosensors. A mobile laboratory-based ELISA that fully replicates the functions of a benchtop ELISA was tested in the field [66]. The mobile ELISA collects whole blood by pricking a finger and simultaneously detects HIV antibodies, treponemal antibodies for syphilis, and non-treponemal antibodies for active syphilis infections. A sample of 96 individuals were tested, with 97% stating a preference for the mobile phone-based sensor compared to the conventional testing method. Additionally, 95% preferred finger-prick blood collection over the typical venipuncture method.

**Figure 5:**
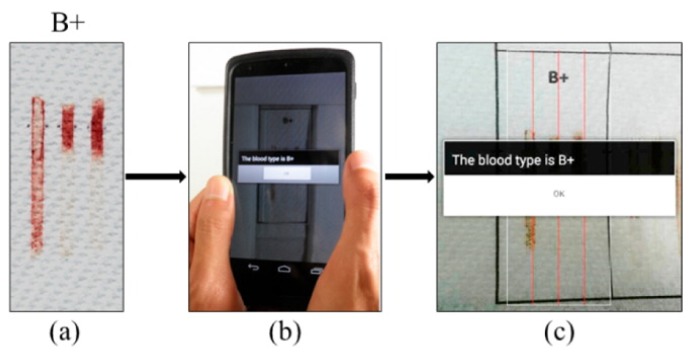
Blood type detection using smartphone. (**a**) Paper sensor with blood-tracks visible in channels. (**b**) Smartphone app developed for interpreting the sensor’s result. (**c**) Detected blood type is displayed as text. Reprint adapted with permission from [67]. Copyright 2018 American Chemical Society.

In addition to detection of infectious diseases, responders to outbreaks require access to standard medical tests in the field. To fulfill this demand a paper-based blood type detector is developed in Guan et al. (2014) [67] (Figure 5). In this sensor, hydrophobic channels treated with Anti-A, -B, and -D antibodies were printed on a paper substrate. Blood reacts differently in each channel according to its blood type. A visible difference of the different reactions is the eluting lengths of blood in each channel. An accompanying app, installed on a smartphone, photographs the paper sensor and, in a similar way to that of a bar code, analyses the lengths of the bars to detect the blood type. The blood type is reported to the user in the form of a text message. It was reported that all 8 ABO/RhD blood types were detectable.

Information from diverse sensors can be most easily integrated if the sensors interface has a common platform such as a smartphone. Recent sensor advances have either used smartphones as a platform for sensor development or included developed technology to rapidly transfer results to a smartphone [68,69]. Additionally, larger portable systems (that could be deployed in a public health emergency) have wireless communication abilities already integrated and could communicate directly with sensor networks.

The data collected from various sensors may be diverse and will not necessarily translate easily across platforms. However, even simple reporting of qualitative identification (species present or not present) along with geospatial information could provide a wealth of information about epidemic outbreak and containment when collected across a large number of sensors. In addition to hand-held strategies, the network could also include autonomous sensors (such as continuous air-sampling from drone-mounted mass-spec, or stationary surveillance systems) [63,70,71].

### 5.2. Auxiliary Hardware for Mobile Phone-Based POCT

The application of mobile phones in point-of-care systems could be further enhanced by integration of a variety of peripheral sensing platforms and achieve a level of performance which is rival to that of conventional laboratory equipment. Such platforms can be categorized based on their sensing scheme as: Optosensors; Electrochemical sensors; and Mechanical sensors.

Optosensors typically integrate a smartphone camera with additional optical and mechanical components, such as diffraction gratings, optical filters and lenses, optical fibers, and 3D printed mounting cradles. Optosensors allow light intensity quantification, colorimetry, and spectrometry to be performed. Lateral flow assays (LFA) [72] and microtiter plates are widely used for loading samples in such sensors and providing a medium for desired reactions. The detection of serum ferritin (a biomarker for Iron deficiency) has been demonstrated by placing a drop of blood on a disposable AuNP labeled LFA and feeding this into a phone accessory. This was possible by suppression of ambient light, uniform illumination from LEDs, focused images of the test, and capturing of images which are then processed via mobile application [73]. Human trial results of this configuration has demonstrated that the approach is consistent with the laboratory standards for detection of serum ferritin. A smartphone integrated instrument for performing transmission, reflection, and intensity spectroscopy has been demonstrated for detection of biomarkers related to pre-term birth in pregnant women and phenylketonuria [74]. This approach uses: two light sources (white from phone LED and green from a laser diode) for illuminating liquid samples; fiber optics and mirrors for guiding optical beams; and a diffraction grating coupled with the phone camera for generating spectra from recorded images and videos, all of which are mounted on a 3D printed cradle and costing approximately $550. Different samples are placed into liquid compartments of a microfluidic cartridge and the cartridge is swiped through an opening in the cradle while the spectra of each sample are measured through processing the recorded video from a smartphone camera. The developed system demonstrated comparable results to that of a clinic grade instrument and it offers the possibility of multiple and rapid measurement of a wide array of samples.

Electrochemical Sensors involve redox (reduction–oxidation) reactions that can be detected, amplified, and translated into meaningful measurements of biomarker concentration [75]. In this regard, sensing platforms that are interfaced with smartphones for power and data transmission have been developed; moreover, by integrating novel microneedle designs, enabled by soft lithography [76], continuous monitoring of biomolecules and chemicals such as glucose [77] is realized. In Min et al. (2018) [78], a $50 prototype for rapid sepsis detection is developed and tested that relies on measuring cytokine interleukin-3 (IL-3) concentration, which offers more than 5× speed and 10× sensitivity improvement over the current detection gold standard. By capturing IL-3 on magnetic beads (2.7 μm in diameter), which allows direct and fast IL-3 extraction from blood and labelling them for electrochemical reaction, IL-3 detection sensitivity of less than 10 pg/mL is achieved through electrical current measurement. This is then fed to a smartphone via Bluetooth for data storage, cloud upload, and system control.

Developments in mechanical sensing platforms include integrated MEMS structures. These perform stress and mass sensing by means of adsorption of biomolecules/particles (by a receptor-coated free standing structure), and recording the corresponding bending/shift in resonance frequency, which is then translated into concentration of particles. Additionally, by transducing acoustic wave propagation and reflection of a medium into electrical signals, properties of that medium can be characterized. A cheap handheld ultrasound tool “Butterfly iQ” has recently passed FDA approval for 13 clinical applications. This tool uses an array of tunable capacitive transducers [79] instead of piezoelectric ceramics, which are widely used in conventional ultrasound tools. The tool is connected to an iphone with an artificial intelligence component used for image acquisition and analysis. As the tool becomes more widely used by physicians then the potential for improvement in the neural network models increases, thus allowing more reliable diagnosis.

## 6. Data Analytics for POCT

### 6.1. Data Management

Advancement of medical science has resulted in an increase of both the number of conditions the average patient is treated for and the complexity of treatment techniques. As a result, a typical patient may simultaneously receive treatment for different conditions from different practices. For example, the average patient with five or more conditions on Medicare (a US government insurance program) will be treated by 13 outpatient physicians and fill 50 prescriptions per annum [80]. This degree of complexity creates challenges for patients, physicians, and researchers that cannot be adequately tackled by the traditional usage of paper records and archives. With an emphasis on streamlined cross-provider data collection, storage, access and analysis, Electronic Health Records (EHR) have emerged as an appropriate response to the increased demands/requirements of medical treatment. Despite the increasing global adoption of EHR, protocols, and structure, the scope of EHR systems is continuously evolving. Ideally, an EHR system should have the following features [81]:

1. Store and allow access to comprehensive health data including the medical history of the patient;
2. Harmonize with the workflow of health organizations and provide efficient interaction experience;
3. Assist in administrative tasks such as billing, insurance claim filing, and scheduling;
4. Allow efficient access and assist in statistical analysis of data.

The successful implementation of any EHR system necessitates adoption of standards for collection, storage, access, and transfer of medical data. These standards can be broadly categorized into medical standardization and technical standardization. Medical standardization is the employment of universally accepted medical terms, abbreviations, writing format, etc. Standardization of medical concepts and procedures enables improved performance of medical professionals in transfer, referral, and supportive care of patients [82]. A major effort in creating a standard definition of medical concepts and procedures was conducted by the European Committee for Standardization (CEN) and resulted in the creation of the System of Concepts to Support Continuity of Care, commonly referred to as ContSys (EN ISO 13940n). In a more targeted approach, the World Health Organization (WHO) has created a medical classification list that assigns codes to diseases, signs and symptoms, etc. The latest edition, ICD-10, contains more than 14,400 unique codes. This coding system is primarily intended to facilitate statistical international health surveys [82]. Technical standardization is the employment of software and hardware protocols and practices which enable the easy cross-system transfer and access of medical data. Transfer of data between medical facilities that may or may not be part of the same EHR system requires the establishment of standard protocols for communication, messaging, data structures, privacy, and security [82]. To meet these needs, various organizations have proposed different hardware and software standards for EHR systems. The international Organization for Standardization (ISO) has proposed the adoption of ISO/TS 18308 for EHR systems. Key features include interoperability with other EHR systems, storage of both structured and unstructured data, cross-software and hardware platform compatibility, storage of both administrative and clinical data, possibility of translation of stored information to different languages, and reliability data storage. In addition, ISO/TS 18308 mandates recording the chronology of actions by users as means of accountability and requires measures to ensure security and privacy by mandating user identification and different levels of access among other measures.

In another approach to standardization, organizations such as Health Level Seven (HL7) have focused on developing clinical document formats irrespective of the overall data structure. An example is the Clinical Document Architecture (CDA), a widely adopted standard format based on XML and developed by HL7 with the goal of specifying structures and semantics of healthcare documents.

### 6.2. Data-Driven Decision-Making Using Machine Learning Techniques

EHR systems allow efficient access and assist in the statistical analysis of data. Practical decision-making tasks in the context of point-of-care diagnostics can be supported by recent AI/machine learning techniques. In particular, supervised machine learning provides statistical decision models for classification problems, i.e., identifying to which set of known categories (classes) a new observation belongs. The decision model is learned by using training data that are labeled *a priori* with the true class membership. In the following paragraphs, we give a brief introduction to machine learning techniques. Concrete application scenarios and case studies will be discussed in the next subsections. Formally, patterns (objects) to be classified are typically represented by vectors of features. Given the feature space ${ℜ}^{d}$ (i.e., *d* features), the classifier is defined as a mapping function $h:\;{ℜ}^{d}\to C$, where $C=\{C_{1},C_{2},\ldots ,C_{K}\}$ is the set of *K* pre-specified classes. Machine learning methods use a training set $\left\{\left(x_{1},y_{1}\right),\left(x_{2},y_{2}\right),\ldots ,\left(x_{n},y_{n}\right)\right\}$ to automatically learn a classification model *h*, where each training sample $x_{i}\in {ℜ}^{d}$ is associated with a class label $y_{i}\in C,\;i=1,2,\ldots ,n$. Machine learning has a long history and remains a vivid research field, providing a vast number of powerful classification methods [83,84]. The standard pipeline for pattern classification is to represent patterns in terms of features and then apply a suitable classification method. The most popular classification methods include the simple k–nearest-neighbor rule, linear discriminant function, Bayes classification, decision tree, support vector machine, and the family of deep learning (neural networks).

Unfortunately, there are no context- or problem-independent reasons to favor one classification method over another, although it is possible to make recommendations in practice [85]. Generally, each classification method has its advantages and disadvantages for a particular use case. Therefore, combining multiple classification methods can help to compensate the erroneous decisions of one classifier by other classifiers. Practically, such *ensemble methods* turn out to be an effective means of improving the classification performance in many applications [86,87]. A prominent representative of ensemble classification is the random forest approach [88], which uses a decision tree as the base classifier and has demonstrated superior performance in many fields, in particular in computer vision [89]. For instance, the highly successful system for real-time human pose recognition built in Microsoft’s Xbox [90] is based on this approach.

Traditionally, hand-crafted features are used for classification, which requires considerable domain expertise and careful engineering. The recent development of *deep learning* [91,92,93] is currently very promising on automatic feature learning. In fact, a hierarchy of features can be learned to build representations of patterns with multiple levels of abstraction. For understanding images, for instance, the first layer typically learns low-level features like edges in the image. The second layer detects middle-level motifs by spotting particular arrangements of edges. Then, the next layer may assemble motifs into larger combinations that correspond to object parts, and subsequent layers would detect objects as combinations of these parts. Importantly, these layers of features are not designed by engineers, but learned instead from data using a learning procedure. In essence, deep learning methods are representation-learning methods with multiple levels of representation.

Most applications of deep learning use deep convolutional neural networks (CNN). Such architectures are among the most promising and have brought breakthroughs in processing images, video, speech and audio [94] and today, it is widely adopted by the computer vision community [95,96]. Additional architectures include recurrent neural networks, which are particularly useful for processing sequential data such as text and speech. One important further development is to augment these networks with an explicit memory, e.g., by means of the long short-term memory (LSTM) [97] that uses special hidden units, the natural behaviour of which is to remember inputs for a long time. In addition to neural networks, deep learning can also be realized within other frameworks. For instance, deep forests with a decision tree ensemble is proposed in Zhou et al. (2017) [98], which shows performance highly competitive to deep neural networks in a broad range of tasks.

Several deep learning libraries are out there, supporting to build deep learning algorithms of considerable complexity. Particularly popular are, for instance, Tensorflow, Keras, Caffe, and Torch, mostly using Python [99]. Many practical recommendations are given in [100] for successfully and efficiently training and debugging large-scale and deep neural networks. Despite the existence of these deep learning libraries, there is still a lack of comprehensible and easy-to-use high-level tools for the design, training, and testing of deep neural networks. A step towards such tools is the recently proposed system Barista [101], an open-source graphical high-level interface for the Caffe deep learning library.

### 6.3. Application Scenarios

AI/machine learning today offers huge potentials for rapid disease diagnosis. There is an opportunity to use available equipment within hospitals with a universal interface to access a wide range of data. The use of open access data for new/unknown or emerging rare infectious diseases could be used for earlier detection.

In dealing with epidemics within LMIC, the data would be acquired through support pathologists and response teams. Non-invasive and minimally-invasive tactics both enable a suitable entry point. They facilitate the examinations of the dead while respecting attendant considerations, including increasing public acceptance [102]. Utilizing this methodology, diagnosis can be forthcoming thus obviating contamination and spread of disease in poor or remote regions, for example, the recent Ebola response in Africa (e.g., opposition of invasive autopsy due to traditional or cultural beliefs) [103,104].

Worldwide, many infections remain undiagnosed and untreated or are diagnosed at a frustratingly later stage, due to poor diagnostic tools. AI generally and machine learning specifically have here enormous capacities to contribute to solutions for these challenges (for the differences between AI/machine learning see [105]). In particular, formulating machine learning algorithms, to work with infectious diseases, leads to important innovations in diagnostics for epidemic response and prevention. This is of both national and international interest.

A contemporary example, employing an automatic machine learning approach, has the ability to identify real-world latent infectious diseases. This is done by withdrawing substantial data from social media [106], based on sentiment analysis [107].

However, a serious problem with all automatic machine learning approaches (also modern in-depth learning approaches [108]) is that they need (1) very large data sets and (2) top-quality data sets. Often, and particularly in the application scenarios described above we do not have this “big data” and the data available is frequently of insufficient quality and most of all we are dealing with difficult problems, which calls for human-in-the-loop approaches to understand the underlying explanatory factors of the data [109]. Moreover, worldwide there is an increasing need for making results re-traceable, transparent and understandable to the medical domain expert (who rarely is a computer expert). Most of all, if human intelligence is augmented by AI/machine learning methods (and in some cases even overruled), a human expert should still be able to re-enact the machine decision process and understand the context in the local problem setting of the expert [110], thus there is a requirement for application of visual analytical methods properly suited to the medical domain experts in their particular context and problem setting [111]. This is of vital importance when dealing with the aforementioned medical issues and particularly on mobile devices. To move things forward, more cross-domain collaboration is needed involving epidemic response experts. They can provide more recognized infectious diseases, since such samples are still rare in developed regions as in the western countries.

AI/machine learning enhanced solutions will become important, because they can provide faster diagnosis, which will enable a faster and better response to outbreaks. Response workers will know with which virus or bacterium they are dealing. Perfecting, and making research data globally-open-access, is certainly of invaluable importance to response teams in remote regions. It avoids the time consuming need for sending samples to reference labs (e.g., CDC: Centers for Disease Control and Prevention in Atlanta). This can take days or even weeks for results to surface. This is also critically delaying epidemic response; potentially allowing the spread of disease worldwide. A New England Journal of Medicine Article [112] provides a nice overview on currently available digital resources for disease detection.

Globally-open-access data removes the requirement for ethical approval. Researchers can produce open data and publish work on their novel concepts, methods, tools and algorithms. This can be made available to the international research community. In particular, they can apply data augmentation to generate sufficient detail on rare disease data for feeding into sophisticated state-of-the-art methodologies and tools (e.g., deep learning approaches). Blood analysis is essential. Researchers can avoid privacy issues by using federated learning, whereby the data remains local and the machine learning is done via client-side learning [113].

These and other application scenarios also pose technical challenges. One example is the issue with meager amounts of data, where automatic approaches suffer with insufficient training samples. When dealing with rare events (e.g., rare diseases [114]) we are confronted with the problem of class imbalance [115]. In that case, one class represents a circumscribed concept, while the other class represents its counterpart. Consequently, samples from the counterpart class heavily outnumber samples from the positive class. This is, inherently, the case in data sets regarding rare diseases, because there is a large number of patients who do not have that disease [116]. In addition, automatic “black-box” approaches are lacking explicit declarative knowledge representation. Therefore, they have difficulty in generating the required underlying explanatory structures, which are needed for the interpretation of the machine learning results [117]. The absence of explanatory capabilities—particularly in deep learning neural networks—limits the full potential of such systems [118,119]. Here, the interactive machine learning approach [120] will become extremely important; where a human-in-the-loop [121] can help to understand the causality of learned representations, which is gaining importance in the medical domain.

### 6.4. Case Studies

AI/machine learning has seen extensive use as a way of better predicting and supporting rapid intervention in epidemic scenarios. Chakoumakos [122] presented a machine learning system to predict the epidemic potential of each disease outbreak, based on text and geospatial data using the ProMed-mail dataset [123]. Experiments were made using k-nearest neighbor, naïve Bayes, and support vector machine with results reporting the possibility of reducing outbreak reports based on severity with 80% precision and 63.3% recall. Using structured and unstructured hospital data, Chen et al. [123] explore the use of Convolutional Neural Networks (CNN) for multimodal disease risk prediction. This work is particularly interesting, as it attempts to streamline machine learning algorithms for prediction of chronic disease outbreak even with incomplete medical data quality. According to the authors, their approach leads to an 94.8% prediction accuracy, with faster convergence speed using unimodal algorithms.

Due to its recurrent nature, influenza has received special attention in what concerns the use of AI/machine learning techniques. Based on free-text reports from emergency departments, Pineda et al. [124] compare seven machine learning classifiers with an expert-built Bayesian classifier, in the task of improving influenza detection in real time. As reported by the authors, machine learning combined with Natural Language Processing (NLP) (used to categorize influenza-related findings) has great potential for the detection of infectious diseases. In Libbrecht and Noble [125], an overview is provided showing the use of several machine learning algorithms with DNA and RNA sequencing; the authors present multiple considerations regarding the use of supervised, unsupervised and semi-supervised learning methods. Under the hypothesis that machine learning techniques can contribute to identify genetic variations in rapidly evolving pathogens, Holman and Gabuzda [126] developed an machine learning pipeline that uses amino acid signatures in the HIV viral envelop together with a C4.5 decision tree and the PART rule-learning engine to analyze the genetics of rapidly evolving pathogens. The method showed a 75% predictive accuracy and identified multiple signatures associated with HAD diagnostics.

A very interesting case study is lensless blood cell counting: Huang et al. [127] developed a system integrating a microfluidic channel and a CMOS image sensor, and improved its limited resolution from the system-level using super-resolution (SR) processing based on the Extreme Learning Machine-based SR (ELMSR) and Convolutional Neural Network-based SR (CNNSR). Their cell counting results matched well with those of commercial (expensive) flow cytometers.

In addition to machine learning, late-breaking research using AI in neurodegenerative diseases has also shown techniques with potential application to epidemics. Recent work described by Bakkar et al. [128] uses commercial IBM Watson text mining technology to study the alterations of RNA-binding proteins (RBPs) in Amyotrophic Lateral Sclerosis (ALS). The authors were able to find significant alterations in 5 RBPs previously unliked to ALS when compared to controls. Some AI techniques applied to epidemics are already seeing their way through to commercial applications and real-world deployment, as shown by a recently announced microscope that uses deep learning algorithms to automatically identify and count malaria parasites in a blood smear [129]. Forecasting is also an area where AI plays a major role. In Liao et al. [130], the authors propose a method that uses Bayesian belief networks to assess disease outbreak risks at different special scales, based on cases or virus detection rates, concluding that their AI-based method is more accurate than traditional approaches.

## 7. POCT for Clinical Diagnostics within Lower Middle Income Countries (LMICs) and Least Developed Countries (LDCs)

### 7.1. Overall Framework

A major opportunity for POCT connectivity and analytics within LMICs and LDCs relates to infectious disease diagnosis and disease management. Smartphone-connected diagnostic tests with geo-referencing information could help prevent the spread of epidemics and transform the approach to global health. POCT could facilitate rapid diagnosis from simple blood pin-pricks or non-invasive swabs. Technologies currently under development aim for an operator without specialized training to safely collect and test a sample in the field within minutes. In brief, this provides faster confirmations without the necessity to send many samples to reference labs such as the CDC Atlanta, avoiding a process that can take days or even weeks before results are reported (delaying the response to outbreaks). While we highlight recent advances in POCT for infectious diseases below, we note that given the increase of non-communicable diseases within LMICs and LDCs—cardiovascular diseases and cancer have been the two highest causes of mortality since 2001 [131]—there is also very significant potential for use of POCT within these disease areas.

Advances in portable diagnostic technology have enabled multiple methods of pathogen detection that can be employed at (or close to) the point-of-care [132,133]. Rapidly deployable, mobile labs [134] and handheld sensor units can provide rapid pathogen identification and confirmation. Connecting results from a wide array of these tests with geospatial information could enable more rapid outbreak detection and a more effective public health response. Such a network of sensors could complement developing national biosurveillance systems [135] and provide a platform for coordinated international response to emerging biothreats. Miniaturised sensing systems that use or connect to a smartphone are of particular interest, as they may be widely deployed and be more broadly integrated into disease-monitoring networks [68]. Analytical techniques that may be integrated with a smartphone analyzer include nucleotide-based detection strategies (PCR, LAMP) [136,137,138] and antibody-based detection (lateral flow, acoustic wave, or other immunoassay approaches) [2,139,140,141]. While these approaches generally require prior knowledge of the pathogen of interest, assay multiplexing is becoming increasingly feasible [140]. Additionally, portable mass spectrometry and miniaturized sequencing strategies may soon enable portable, novel pathogen detection. In addition to providing pathogen identification or confirmation, portable analytical strategies have recently been combined with rapid incubation or culture methods to provide antibiotic resistance information in as few as 30 min [142]. Rapid detection of developing antibiotic resistance though networked sensors could help ensure proper initial treatment and containment in an epidemic.

### 7.2. Open Data Platforms for Infectious Diseases

Several open data platforms for infectious diseases have been deployed in developed or medium income countries, and may serve as a model for broader open platforms in resource limited areas. Real-time geo-tracking resources, such as Outbreak Near Me [143] utilize user feedback, news outlets, and official reports to track outbreaks in real time. This app was developed by Boston children hospital, Google, and the CDC. In fact, the US federal government has created several open access databases under the umbrella of the National Institute of Health’s Bioinformatics Resource Centers (BRCs) for Infectious Diseases, to assist researchers studying infectious diseases. These efforts are happening globally, at a broader level, showing the relevance of the topic and how political commitment is changing the paradigm in different nations.

To highlight a few examples, the government of Taiwan publishes detailed health statistics in the form of a regularly updated open access database. The database is published in Mandarin Chinese and is reported to contain data on occurrences of 70 different infectious diseases starting from 2004 [144]. The National Infectious Disease Register operated by the Finnish government collects occurrences of infectious diseases in that country. The database is accessible online and has been updated routinely from 1995. Approximately 70 specified microbes as well as all blood and cerebrospinal fluid findings are reported to the infectious diseases register.

### 7.3. Nucleic Acid Amplification Techniques

**Figure 6:**
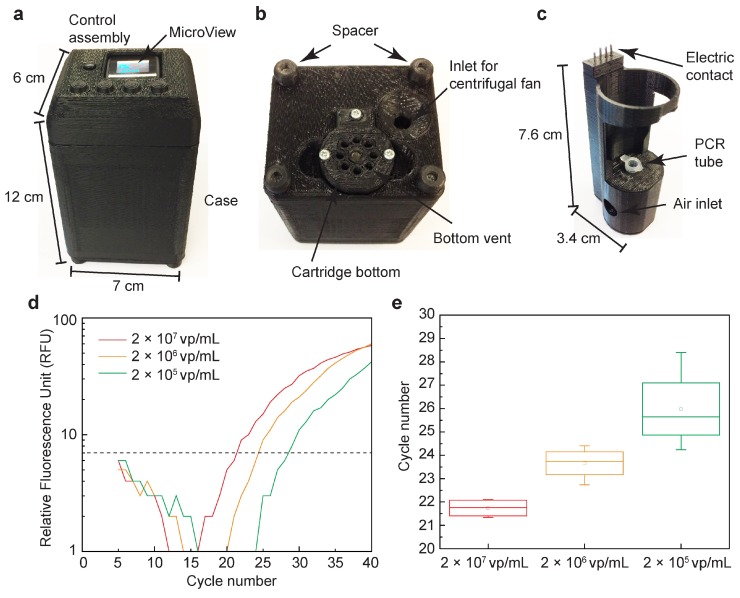
3D printed real-time PCR machine for lentivirus detection and quantification. The small device is capable of quantifying different viral loads using fluorescence measurements of DNA. (**a**) Top view of the qPCR device with MicroView screen showing the status of the amplification cycle and fluorescence reading. (**b**) Bottom view of the qPCR machine displaying cartridge bottom, spacer, and inlet for centrifugal fan. (**c**) Dimensions of the cartridge. (**d**) Fluorescence measurement illustrating change in intensity for different virus concentrations. (**e**) Measured Cq for three concentrations of target DNA. Reprint from [24].

Nucleic acid amplification and sequencing techniques are highly specific, and have recently become more rapid and miniaturized to the point of a practical hand-held device. A battery-operated real-time PCR machine was reported to have a size of 12 × 7 × 6 cm${}^{3}$ and a weight of 214 g, and was capable of detecting the presence of lentivirus and quantify the amount of viral load using quantitative PCR (Figure 6) [24]. Similarly, a handheld real-time PCR device was reported to process 4 PCR samples in about 30 min [145]. GeneXpert systems by Cepheid continue to be the gold standard for point-of-care diagnostics and, they use integrated cartridges to automate the sample preparation, reagent mixing, and quantitative PCR detection [146]. GeneXpert Omni is expected to transform the fully-integrated platform into a point-of-care diagnostic system.

Nucleic acid amplification is typically carried out using PCR, and there remain significant limitations to its use within a POCT device. Traditional PCR requires thermocycling, involving increase power requirements and analysis times. There are now several isothermal amplification approaches, with high sensitivity and specificity. A particularly notable method amenable to POCT device development is the Loop Mediated Isothermal Amplification (LAMP), which detects both DNA and RNA targets [147]. Isothermal amplification has several advantages within a POCT setting including lower power requirements and less harsh conditions for combined immunological assays.

### 7.4. Rapid DNA/RNA Sequencing for Outbreak Response

Rapid gene sequencing technologies aid quick response to disease outbreaks, by identifying the virulence factor and the path of disease transmission [148]. While this technique has generally been applied within centralized or regional laboratories, POC implementations are increasingly possible. Nanopore recording of DNA/RNA translocation is a promising technology in low-cost, rapid sequencing with sampling rates over 10k Samples/s to detect individual base translocations [149]. With a fast sequencing rate, the nanopore-based sequencing has a potential to reduce the detection time, and provide information about sub-strains and therapeutic resistance during an outbreak [150].

**Figure 7:**
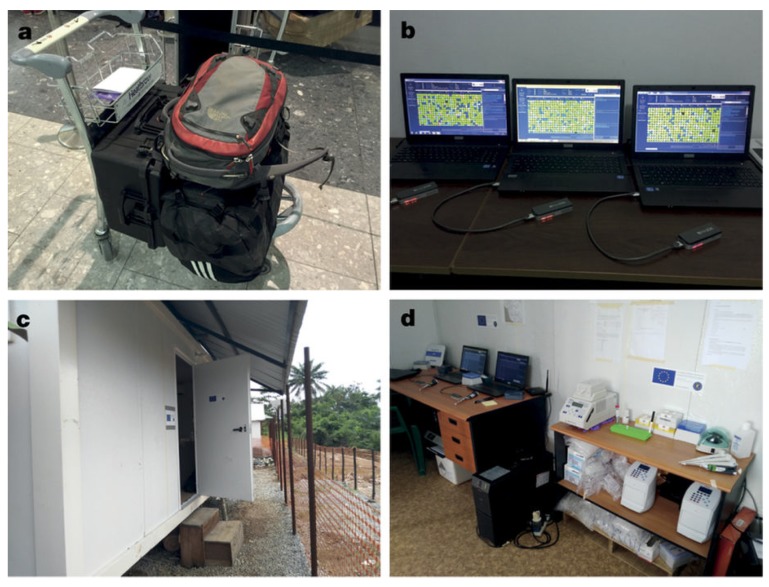
Portable genome sequencing for Ebola surveillance in Guinea. The system consisted of three MinION instruments, four laptops, a thermocycler for PCR, and other tools. Panel (**a**) shows that the authors used aircraft baggage to carry all instruments, reagents and disposable consumables. Panel (**b**) shows that the authors initially established the genomic surveillance laboratory in Donka Hospital, Conakry. Panel (**c**) is used to indicate that they later moved the laboratory to a dedicated sequencing laboratory in Coyah prefecture. Panel (**d**) shows an image of the room inside this laboratory where they separated the sequencing instruments (left side) from the PCR bench (right side). Power to the thermocycler was provided by an un-interruptable power supply, which can be observed in the middle of the room. (Photos were taken by Josh Quick and Sophie Duraffour.) Reprint from [153].

Integrated complementary metal–oxide–semiconductor (CMOS) bioelectronic systems have also been developed, which incorporate the nanopore directly on the CMOS surface to reduce the noise source (input capacitance to transimpedance amplifier) and maximize the sampling rate [149,151]. Using the high-density integration of CMOS integrated circuits, many nanopores can potentially record DNA translocations in parallel, yielding a much higher sequencing rate. This approach has been used in the development of MinION, a pocket-sized nanopore sequencer, by Oxford Nanopore Technologies, which achieves sequencing speeds in the order of 100 nucleotides/s [152]. Recently, a genome sequencing system using MinION was used to support a rapid outbreak response by characterizing the infectious agent in Guinea with the ongoing epidemic [153] (Figure 7).

### 7.5. Antimicrobial Resistance

POCT could play an important role in supporting the management of conditions for individual patients and also provide wider control of STIs within developed countries, LMICs and LDCs. Novel and more effective screening techniques for AMR are particularly relevant in LMICs and LDCs. These countries have the majority of global incidents of STIs, but the health systems are less well resourced to manage them. As an example, gonorrhoea is a significant public health concern with AMR in Neisseria gonorrhoea and so there is an urgent need for new antimicrobials and action for control.

Multiple efforts are being developed around the topic of antimicrobial susceptibility [142]. In Schneider et al. (2017) [154], the authors used a resonant mass method [155] to develop a 96-well plate that provides rapid testing of several antibiotics. The technique provides an array of micro-channels to monitor microbial growth in broth microdilutions, by detecting and measuring the mass of individual microbes. Results have shown that a rapid antimicrobial susceptibility testing (AST) produces correct and repeatable outputs in both lyophilized and freshly prepared liquid antibiotic panels in under three hours.

Also focusing on rapid AST testing, the work by GeneFluidics Inc. (2019) [156] has resulted in a fully automated system that uses a reagent kit and disposable sensor array to quantify species-specific 16S ribosomal RNA (rRNA). An approach based on multi-channel potentiostat measurement for the enzymatic cycling amplification is used.

The applicability of POCT can be greatly enhanced if the testing procedures are made available in a portable fashion. Aligned with such goal, in Andreyev et al. (2017) [157] the authors propose a method for hand-held molecular diagnostics. In their proposed approach, an electronic module based on polymerase chain reaction is reportedly able to use a reagent to detect the presence of a target amplicon within an input sample.

Reflecting the urgent need for countermeasures to the potential development and rapid global spread of infectious diseases, the United States’ National Health Institute (NIH) and Biomedical Advanced Research and Development Authority (BARDA) jointly held a $20 million contest challenging groups to develop rapid POC diagnostic tests for antimicrobial resistant bacteria. The AMR challenge finalists are given in Table 2. The approaches that are adopted are likely to include advances in transduction mechanisms, microfluidics and use of machine learning to distinguish between viral and microbial infections, as well as the development of assay capable of identifying drug resistant bacteria.

## 8. Final Considerations and Future Outlook

All countries face a gap between the funding that is needed to support healthcare infrastructure and the amount of funding that is actually available to meet these needs. This gap can in part be addressed through the use of connected POCT devices with AI/machine learning for enhanced sensing. This offers the huge possibility for earlier identification of disease where treatment is likely to be more effective as compared to a patient with symptomatic disease at a late stage. It also offers the urgently needed possibility of delivering care in a decentralised way within primary and tertiary care settings. In the context of global health within LMICs and LDCs, this can be particularly important in preventing the spread of epidemics. Realisation of this vision will require considerable inter-disciplinary effort including in: chemistry, materials, biology, electronics, mechanics, micro and nanotechnologies, photonics, biotechnology, and particularly AI/machine learning. There are likely to be continued innovation in microfluidics, transducers, chemistry/biology, and AI/machine learning, which will help to unlock the full potential of POCT to transform testing and improve human health. The authors of this paper are dedicated to support these cross-domain efforts on an international level for the benefit of people worldwide.

## Figures and Tables

**Table 1:** Biomarkers.

Cancer Biomarkers	Biomarker	Normal Values in Blood
	PSA	4 ng/mL
	IL-6	6 pg/mL
	IL-8	13–20 pg/mL
	MMP-2	367–770 ng/mL
	MMP-3	15–72 ng/mL
	Alpha-fetoprotein	<20 ng/mL
	CEA	5 ng/mL
	CA-125	35 U/mL
**Cardiac Biomarkers**	CRP	3 mg/mL
	NT-proBNP	1 ng/mL
	CTnT	0.3 ng/mL
	CTnl	0.01–0.1 ng/mL
	Myoglobin	50–100 ng/mL

**Table 2:** AMR Challenge Finalists.

Title	Analytic Approach	Sample Type	Result and Time	Format
Ultra-Rapid Infection Confirmation and Phenotypical AST by Microbe Mass Measurement [http://www.lifescaleinstruments.com/ https://patents.google.com/patent/US20150072373A1/en]	Microfluidic, mass detection of bacterial growth	blood culture, urine culture demonstrated (extension to urine screen, cerebral spinal fluid, pleural fluid planned)	Phenotypic antibiotic resistance (minimum inhibitory concentration) Time to result: 3–3.5 h	desktop device
Single Cell Biometric Analysis for Rapid ID/AST [http://klarisdx.com/ https://patents.google.com/patent/US20180172675A1/en]	Microfluidic partitioning of single cells; detection of phenotypic antimicrobial susceptibility with redox-sensitive viability dye	not specified	Phenotypic antibiotic resistance, pathogen ID Time to result: 4 h	desktop device
Fully Automated Pathogen ID and AST Directly from Blood and Urine [http://www.genefluidics-lifescience.com/]	Electrochemical sandwich hybridization of 16S ribosomal RNA	unprocessed urine demonstrated, (whole blood in development)	Pathogen genus/species by 16S homology Time to result: 30 min for pathogen ID, 90 min for resistance profile (for urine)	desktop device with disposable sensor array chip
Patient-side, Disposable, Molecular PCR Diagnostic Device for Neisseria Gonorrhea and Drug Resistance Markers [https://www.sbir.gov/sbirsearch/detail/1323659 https://patents.google.com/patent/US9623415B2/en]	Miniaturised PCR	genital tract swab	Pathogen ID, (ciprofloxacin resistance in development) Time to result: 25 min	single-use, disposable POC device
Host Gene Expression to Classify Viral and Bacterial Infection Using Rapid Multiplex PCR [https://www.predigen.com/] Journal of Clinical Microbiology Jan 2010, 48 (1) 26-33; DOI: 10.1128/JCM.01447-09	PCR of host gene expression patterns	blood	confirmation of viral-type host response pattern, determination of viral/bacterial co-infection Time to result: 45 min	desktop/multiplex PCR equipment
